# Clinicopathological characterisation of MTAP alterations in gastrointestinal cancers

**DOI:** 10.1136/jcp-2023-209341

**Published:** 2024-02-13

**Authors:** Gianluca Mauri, Giorgio Patelli, Laura Roazzi, Emanuele Valtorta, Alessio Amatu, Giovanna Marrapese, Erica Bonazzina, Federica Tosi, Katia Bencardino, Gabriele Ciarlo, Elisa Mariella, Silvia Marsoni, Alberto Bardelli, Emanuela Bonoldi, Andrea Sartore-Bianchi, Salvatore Siena

**Affiliations:** 1 IFOM ETS – The AIRC Institute of Molecular Oncology, Milan, Italy; 2 Department of Oncology and Hemato-Oncology, University of Milan, Milan, Italy; 3 Department of Hematology, Oncology, and Molecular Medicine, Grande Ospedale Metropolitano Niguarda, Milan, Italy; 4 Department of Pathology, Grande Ospedale Metropolitano Niguarda, Milan, Italy; 5 Department of Oncology, Molecular Biotechnology Center, University of Torino, Turin, Italy; 6 Division of Research and Innovation, Department of Hematology, Oncology, and Molecular Medicine, Grande Ospedale Metropolitano Niguarda, Milan, Italy

**Keywords:** Gastrointestinal Neoplasms, Pathology, Molecular

## Abstract

**Background:**

Methylthioadenosine phosphorylase (MTAP) is an essential metabolic enzyme in the purine and methionine salvage pathway. In cancer, *MTAP* gene copy number loss (*MTAP* loss) confers a selective dependency on the related protein arginine methyltransferase 5. The impact of *MTAP* alterations in gastrointestinal (GI) cancers remains unknown although hypothetically druggable. Here, we aim to investigate the prevalence, clinicopathological features and prognosis of *MTAP* loss GI cancers.

**Methods:**

Cases with *MTAP* alterations were retrieved from The Cancer Genome Atlas (TCGA) and a real-world cohort of GI cancers profiled by next-generation sequencing. If *MTAP* alterations other than loss were found, immunohistochemistry was performed. Finally, we set a case–control study to assess *MTAP* loss prognostic impact.

**Results:**

Findings across the TCGA dataset (N=1363 patients) and our cohort (N=508) were consistent. Gene loss was the most common *MTAP* alteration (9.4%), mostly co-occurring with *CDKN2A/B* loss (97.7%). Biliopancreatic and gastro-oesophageal cancers had the highest prevalence of *MTAP* loss (20.5% and 12.7%, respectively), being mostly microsatellite stable (99.2%). In colorectal cancer, *MTAP* loss was rare (1.1%), while most *MTAP* alterations were mutations (5/7, 71.4%); among the latter, only *MTAP-CDKN2B* truncation led to protein loss, thus potentially actionable. *MTAP* loss did not confer worse prognosis.

**Conclusions:**

*MTAP* alterations are found in 5%–10% of GI cancers, most frequently biliopancreatic and gastro-oesophageal. *MTAP* loss is the most common alteration, identified almost exclusively in MSS, *CDKN2A/B* loss, upper-GI cancers. Other *MTAP* alterations were found in colorectal cancer, but unlikely to cause protein loss and drug susceptibility.

**WHAT IS ALREADY KNOWN ON THIS TOPIC**

- Despite being potentially druggable, the impact of methylthioadenosine phosphorylase (*MTAP*) alterations in gastrointestinal (GI) cancers is still largely unknown.

**WHAT THIS STUDY ADDS**

- Gene loss is the most common *MTAP* alteration, almost exclusively occurring in upper-GI, microsatellite stable, *CDKN2A/B* loss cancers. *MTAP* loss in GI cancers did not impact patients’ prognostic. In colorectal cancer, *MTAP* alterations other than gene loss were found, but they were not associated to protein loss, thus unlikely druggable in ongoing trials.

**HOW THIS STUDY MIGHT AFFECT RESEARCH, PRACTICE OR POLICY**

- Our study provides the largest clinicopathological and prognostic characterisation of *MTAP* altered GI cancers. This analysis will be instrumental in refining patients selection for clinical trials harnessing *MTAP* alterations.

## Introduction

 The mechanism of action of most targeted anticancer therapies is essentially the inhibition of oncogenic stimuli through tyrosine-kinase receptor blockade.^1^,^3^ However, oncogene-addicted cancers eventually develop resistance under drug-induced selective pressure, limiting targeted therapy efficacy.^4^ Thus, alternative or complementary therapeutic approaches are warranted.

Antimetabolic targeted therapies are emerging as new strategies to induce apoptosis and cell death in cancer, given that the pharmacological restraining of certain nutrients or metabolic substrates has shown antitumour activity in preclinical models.^5^ In this context, polyamine biosynthesis is garnering interest for its role in different cancer types.^6^

The *MTAP* (methylthioadenosine phosphorylase) gene encodes for an enzyme that plays a key role in polyamine metabolism and the salvage pathway of purines and methionine.^7^ This gene is located in the human chromosome 9p21.3, close to cyclin-dependent kinase 2A and 2B (*CDKN2A* and *CDKN2B*), which are two well-known oncosuppressors involved in the regulation of the cell cycle.^8^ *MTAP* is often codeleted with *CDKN2A/B* in a variety of cancers through chromosome 9p21.3 microdeletion events. While *MTAP* loss was initially alleged as a ‘passenger’ genomic event within the broader context of contiguous tumour suppressor gene loss, recent findings suggest it may also play an independent role in carcinogenesis.^9 10^ Indeed, preclinical studies showed that *MTAP* loss promotes tumourigenesis independently from the presence of *CDKN2A/B* loss.^811^,^13^ Homozygous loss of *MTAP* occurs in around 15% of all human cancers and seems to be associated with a more aggressive phenotype with worse prognosis in different malignancies, such as non-small cell lung cancer.^14^,^16^ Notably, *MTAP* loss is highly prevalent in thoracic cancers, reaching approximately 70% in mesothelioma and 60% in lung adenocarcinoma.^17 18^ Regarding gastrointestinal (GI) cancers, *MTAP* loss prevalence was reported in heterogenous case series often focusing on individual tumour types. It was reported in 30% of pancreatic cancer (PC),^19 20^ 12%–35% of biliary tract cancer (BTC)^21^ and approximately 20% in gastro-oesophageal cancer (GEC),^22 23^ while data are lacking for colorectal cancer (CRC).

**Figure 1:**
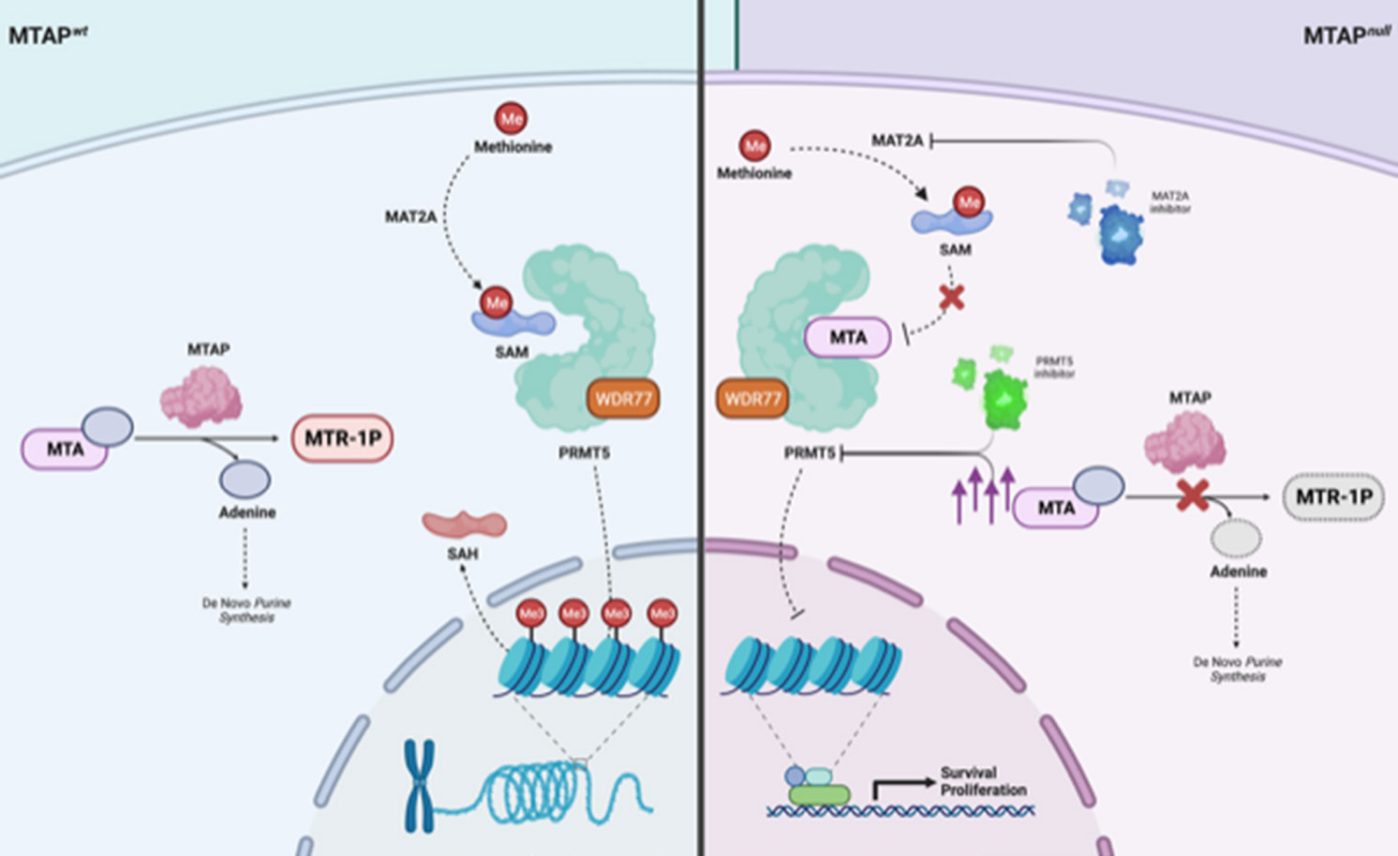
Therapeutic vulnerability of *MTAP* null cells by MAT2A and PRMT5 inhibitors *MTAP* loss results in intracellular accumulation of MTA, which competes with the activating cofactor SAM, causing a decrease in PRMT5 activity. PRMT5 is a key enzyme for the methylation of proproliferative kinases, thus the reduction in PRMT5 activity leads to activation of oncogenic pathways. Targeting MAT2A or PRMT5 in MTAP null tumours is a potential therapeutic strategy. Created with BioRender.com. Me, methyl group; MTA, methylthioadenosine; MTR-1P, 5-methylthioribose-1-phosphate; MTAP, methylthioadenosine phosphorylase; PRMT5, protein arginine methyltransferase 5; SAH, S-adenosyl homocysteine; SAM, methyl donor S-adenosyl-L-methionine; WDR77, WD repeat domain 77; wt, wild type.

The loss of *MTAP* has been identified as a potential therapeutic target in cancer due to its multiple cellular effects.^7 10 24^ In MTAP deficient cells, the accumulation of its substrate methylthioadenosine (MTA)^24^ can hamper the activity of protein arginine methyltransferase 5 (PRMT5) via direct feedback.^10^ MTA, as a structural analogue of the methyl donor S-adenosyl-L-methionine (SAM) required for PRMT5 activity, competes with SAM for binding and can effectively inhibit PRMT5 at high concentrations. PRMT5 plays a pivotal role in the methylation of various cellular substrates, including pro-proliferative kinases, histones and critical transcriptional components. Its activity stimulates cellular proliferation and biosynthesis, as showed in different tumour models. From a pharmacological standpoint, exploiting *MTAP* loss can be approached in at least two distinct ways for two primary reasons: (1) inhibition of de novo purine synthesis and (2) further inhibition of PRMT5, which can be achieved both indirectly, via the accumulation of MTA, and directly through targeted inhibitors. The loss or inhibition of PRMT5 results in altered RNA splicing and an increase in DNA damage, as highlighted in preclinical studies.^24^ Moreover, effective targeting of polyamine biosynthesis has demonstrated potent antiproliferative and prodifferentiating effects in preclinical models^7 10 25^ ^26^ (figure 1).

GI cancers are among the most lethal tumours worldwide, due to a high risk of relapse and metastatisation.^27^ Among others, cancers arising from the biliopancreatic system retain a particularly poor prognosis owing to the early acquisition of resistance to available anticancer therapies. Identifying new exploitable mechanisms to develop effective targeted treatments is one of the most urgent needs in oncology.

In this study, we purport to assess the prevalence, clinicopathological features and prognostic impact of *MTAP* alterations in GI cancers, by leveraging publicly available repositories and integrating data from a patient cohort at our institution.

## Methods

### The Cancer Genome Atlas PanCancer Atlas Analysis

We conducted a retrospective cohort study using data from The Cancer Genome Atlas (TCGA) accessed via the cBioPortal (https://www.cbioportal.org/, accessed on 25 July 2023).^28 29^ The scope of our research was confined to GI tumours, specifically GEC, CRC, BTC and PC.^30^ We queried for patients with unique tumour samples that had been profiled for somatic mutations and copy number alterations (CNA) of *MTAP*, among other genes.

Relevant clinical and molecular characteristics were evaluated using the information provided by the cBioPortal.^28 29^ Further, we narrowed down our investigation to samples exhibiting homozygous *MTAP* loss, achieved by filtering the downloaded tabular list of cases according to ‘*MTAP* homodeletion’ status in the ‘R’ software package (V.2023.06.0+421, The R Foundation). A control group for comparative analysis was formed by subjects with diploid wild-type *MTAP* (*MTAP* unaltered cohort), excluding those patients with mutations or other *MTAP* alterations of unknown significance. We then compared clinical and molecular characteristics, progression-free survival (PFS) and overall survival (OS), between these two groups (*MTAP* loss vs diploid *MTAP* wild type) using the cBioPortal^28 29^ and ‘R’ software.

### Niguarda cohort analysis

From July 2019 to January 2022, we retrospectively collected the results of next-generation sequencing (NGS) analysis obtained through the FoundationOne CDx assay on archival tissue samples of advanced GI tumours at Grande Ospedale Metropolitano Niguarda, Milan, Italy. We included GI tumours only (from oesophagus to anus, liver, pancreas and biliary tract). Samples that failed the NGS analysis due to insufficient or low-quality material were excluded. Data from medical records were annotated on the REDCap platform,^31^ together with the *MTAP* status and other molecular results for each subject with *MTAP* altered tumours, and preserved anonymously with respect to the patients’ privacy. All patients accepted and signed an informed consent for molecular screening through FoundationOne CDx within GO40782/STARTRK-2 trial (NCT02568267).

We further assessed MTAP protein expression by immunohistochemistry (IHC) using a Rabbit polyclonal anti-MTAP antibody (1:200 dilution, ProteinTech, Tucson, Arizona, USA) in selected tumours harbouring *MTAP* alterations, other than *MTAP* loss, to evaluate whether these were associated with loss of protein expression, thus potentially conferring sensitivity to targeted agents currently under clinical investigation in dedicated clinical trials. As previously reported,^32^ we consider as MTAP deficient only those tumours with complete absence of expression in IHC (score 0). Using the same criteria as a control we also evaluated the expression loss of the protein p16 (the product of *CDKN2A*) (p16, Clone:JC2, mouse monoclonal antibody, 1:100 dilution, Gennova, Sevillia, Spain), that it is commonly associated with *MTAP* loss. Immunohistochemistry analyses were performed on formalin-fixed paraffin-embedded tumour sections by an automated staining system Dako Omnis. Normal stromal cells were used as internal positive control.

Lastly, focusing on *MTAP* loss, we set a case–control study comparing GI cancers with *MTAP* loss to *MTAP* unaltered cases in a 1:2 ratio, matched by primary tumour site. While our primary interest was *MTAP* gene loss, cases with other types of *MTAP* alterations were also considered if they exhibited a complete absence of MTAP protein expression, as indicated by an IHC score of 0.

### Statistical analysis

Due to the potential for an inflated risk of false-positive findings from multiple hypothesis testing in the TCGA cohorts, we set a stringent prespecified level of significance at p<0.001 for all statistical analyses. Results were then validated whenever possible in our independent case–control study according to a standard prespecified p<0.05. We used the χ^2^ test for categorical data and the Wilcoxon rank-sum test for numerical data to refute the null hypothesis of no difference in variables between the *MTAP* loss and the *MTAP* unaltered cohorts (except otherwise specified). Numeric variables were expressed as medians/IQR. Median age was calculated at the time of cancer diagnosis. For survival data (ie, time to progression or censoring for PFS and time to death or censoring for OS), we employed the log-rank test to refute the null hypothesis of no difference in survival rates between the two independent cohorts. PFS and OS data from the TCGA cohorts previously proved reliable and were recommended for use.^33^ All statistical analyses were executed in ‘R’ (V.2023.06.0+421, The R Foundation), except for those readily computable directly in the cBioPortal.^28 29^

## Results

### TCGA PanCancer Atlas Analysis

An initial cohort of 1436 GI cancers was identified through cBioPortal based on the study inclusion criteria. Of these, 73 patients were excluded since the *MTAP* status had not been profiled, resulting in a selected cohort of 1363 patients. Since *MTAP* is a tumour suppressor gene reported to promote tumour growth by copy number loss,^10^ we then focused on 128 cases exhibiting *MTAP* loss (*MTAP* loss cohort). For comparison, we also established a control cohort comprising 1224 patients who showed no *MTAP* alteration (*MTAP* unaltered cohort) (online supplemental figure 1A).

We first explored the prevalence of *MTAP* gene alterations in the overall cohort of GI cancers (N=1363), together with clinical and molecular characteristics (online supplemental table 1). This miscellaneous cohort of patients with GI cancer, primarily non-metastatic, exhibited *MTAP* alterations in 10.3% of cases (N=139), most commonly as copy number loss (9.4%, N=128), followed by mutations (0.6%, N=7) and amplification (0.3%, N=4). Clinical and molecular characteristics of *MTAP* loss, *MTAP* mutant and *MTAP* amplified GI tumours are presented in table 1.

**Table 1:** Clinical and molecular characteristics of GI malignancies exhibiting *MTAP* alterations, presented as gene loss, mutations and amplification in the TCGA PanCancer Atlas Studies and the Niguarda Cancer Center cohort

	TCGA cohort (N=135)			Niguarda cohort (N=27)	
	*MTAP* loss	*MTAP* mutant	*MTAP* amplified	*MTAP* loss	*MTAP* mutant
No of patients	128	7	4	22	5
Median age (IQR)	63 (56–72)	71 (62–72)	62 (53.72)	62 (48–72)	55 (47–68)
Gender (%)					
Male	89 (69.5)	1 (14.3)	3 (75.0)	13 (59.1)	3 (60.0)
Female	39 (30.5)	6 (85.7)	1 (25.0)	9 (40.9)	2 (40.0)
Cancer type (%)					
Pancreas	40 (31.3)	0 (0.0)	1 (25.0)	12 (54.5)	0 (0.0)
Gastro-oesophageal	78 (60.9)	3 (42.9)	1 (25.0)	4 (18.2)	0 (0.0)
Colorectal	6 (4.7)	4 (57.1)	2 (50.0)	2 (9.1)*	5 (100.0)
Biliary tract	4 (3.1)	0 (0.0)	0 (0.0)	2 (9.1)	0 (0.0)
Others	0 (0.0)	0 (0.0)	0 (0.0)	2 (9.1)	0 (0.0)
Tumour histology (%)					
Adenocarcinoma	95 (74.2)	5 (71.4)	4 (100.0)	19 (86.4)	5 (100.0)†
Mucinous adenocarcinoma	2 (1.6)	0 (0.0)	0 (0.0)	1 (4.5)	0 (0.0)
Signet ring carcinoma	4 (3.1)	1 (14.3)	0 (0.0)	1 (4.5)	0 (0.0)
Squamous carcinoma	27 (21.1)	1 (14.3)	0 (0.0)	0 (0.0)	0 (0.0)
Undifferentiated carcinoma	0 (0.0)	0 (0.0)	0 (0.0)	1 (4.5)	0 (0.0)
Stage at diagnosis (%)					
Non metastatic	82 (64.1)	5 (71.4)	2 (50.0)	9 (40.9)	4 (80.0)
Metastatic	12 (9.4)	1 (14.3)	1 (25.0)	13 (59.1)	1 (20.0)
NA	34 (36.2)	1 (14.3)	1 (25.0)	0 (0.0)	0 (0.0)
Tumour mutational burden-high‡ (%)	4§ (3.1)	4§ (57.1)	0§ (0.0)	1 (4.5)¶	1 (20.0)
Microsatellite instability (%)	1** (0.8)	4** (57.1)	0** (0.0)	0 (0.0)	1 (20.0)
Concomitant *CDKN2A-CDKN2B* deletion (%)	125†† (97.7)	1 (14.3)	1 (25.0)	22 (100.0)	0 (0.0)
Concomitant *MTAP* loss-*RAS* mutations (%)					
Pancreatic	34 (85.0)	NA	1 (100.0)	12 (100.0)	NA
Colorectal	2 (40.0)	3 (75.0)	1 (50.0)	1 (14.3)	1 (20.0)

* Both colorectal cancers were *RAS* and *BRAF* wild type, MSS, with low TMB.

† One adenocarcinoma had squamous foci.

‡ ≥10 mutations/megabase.

§ Non-synonymous TMB.

¶ POLE mutatation found.

** According to the MANTIS score with a threshold of 0.4.

†† *CDKN2A* in 97.7% of cases, while *CDKN2B* in 92.2%. *CDKN2A* loss always reported for all *CDKN2B* loss cases, co-occurrence 118/128 cases. Log2 OR >3, p<0.001 (derived from two-sided Fisher’s exact test).

GIgastrointestinalMANTISMicrosatellite Analysis for Normal-Tumor InStabilityMSSMicrosatellite stableMTAPmethylthioadenosine phosphorylaseNA, not applicableOROdds ratioTCGAThe Cancer Genome AtlasTMBTumour mutational burden

Given that gene deletion was the most common alteration observed for *MTAP* and that its role in cancer is supported by evidence of pathogenicity,^10^ we further focused the analysis on *MTAP* loss cases (N=128). Prevalence of *MTAP* loss events was higher in PC (22.3%), followed by GEC (12.7%) and BTC (11.1%). Conversely, *MTAP* loss was quite rare in CRC (1.1%). While addressing a genetic deletion, we investigated CNA for adjacent genes at the same chromosomal location (9p21.3). We observed nearly a complete overlap of *CDKN2A/B* loss in the *MTAP* loss cohort (97.7%, N=125), with a statistically significant odds ratio (OR) for co-occurrence (p<0.001, two-sided Fisher’s exact test).

Comparative analysis between the *MTAP* loss (N=128) and *MTAP* unaltered (N=1224), cohorts revealed significant differences among several variables (table 2). Enrichment for *MTAP* loss was confirmed in a subset of tumour types, that is, PC and GEC (p<0.001), while CRC was confirmed as far less represented in the *MTAP* loss population as compared with the unaltered one (p<0.001). Interestingly, histology distribution was also different among cases and controls (p<0.001): while adenocarcinoma was similarly represented as the prevalent histology, squamous cell carcinoma cases were fourfold more common in the *MTAP* loss cohort. As a key issue towards result interpretation, we highlight that almost all the reported squamous-cell cancers were oesophageal cancers in this cohort. From a molecular perspective, *MTAP* loss cases were almost exclusively classified as MSS as compared with controls (99.2% vs 86.6%, p<0.001), with a small difference in median TMB without clinical relevance (2.5 vs 3.3 mutations/megabase, p<0.001). *CDKN2A/B* loss was confirmed significantly enriched in the *MTAP* loss population (97.7% vs 8.1%, p<0.001), and the same result applied to the deletion of several other genes located in proximity to chromosome 9p21.3. Indeed, *MIR31HG*, *IFNA1*, *IFNA5*, *IFNA8*, *IFNE*, *LINC01239*, *DMRTA1* and *KLHL9* on the chromosome cytoband 9p21.3 were found deleted in ≥50% of *MTAP* loss cases (compared with <2% in controls, p<0.001). We also observed minor enrichment (deletion in <50% of cases) for other 75 genes spanning from 9p21.1 to 9p24.3 (p<0.001) (online supplemental figure 2). Regarding classical oncogenes and tumour suppressors in GI cancers, we found no difference in *TP53* and *KRAS* mutations, while *APC* mutations were less common (7.1% vs 35.5%, p<0.001) and *ERBB2* amplifications more common (16.4% vs 7.5%, p=0.001) in case of *MTAP* loss, again likely due to the low prevalence of CRC and high prevalence of GEC in this population. Focusing on individual tumour types, the analysis was not powered to identify differences in subpopulations. As such, we could only observe a trend towards a higher incidence of *KRAS* mutations in *MTAP* loss PC as compared with the unaltered counterpart—85.0% (34/40) vs 60.1% (83/138), p=0.003. At the transcriptomic level, there was a significant association between *MTAP* copy number loss and decreased gene expression (online supplemental figure 3).

**Figure 2:**
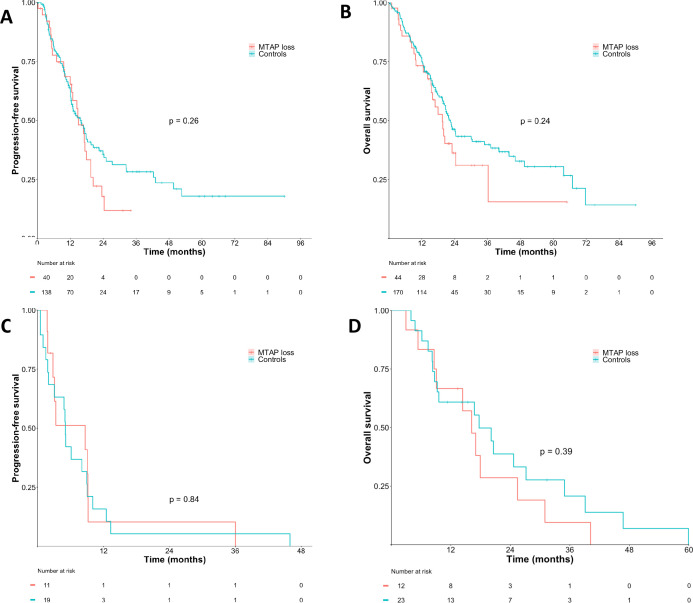
No difference in survival analysis of pancreatic cancer patients according to *MTAP* status in the TCGA PanCancer Atlas cohort (**A, B**) and the Niguarda Cancer Center cohort (**C, D**). MTAP, methylthioadenosine phosphorylase; TCGA, The Cancer Genome Atlas.

**Figure 3:**
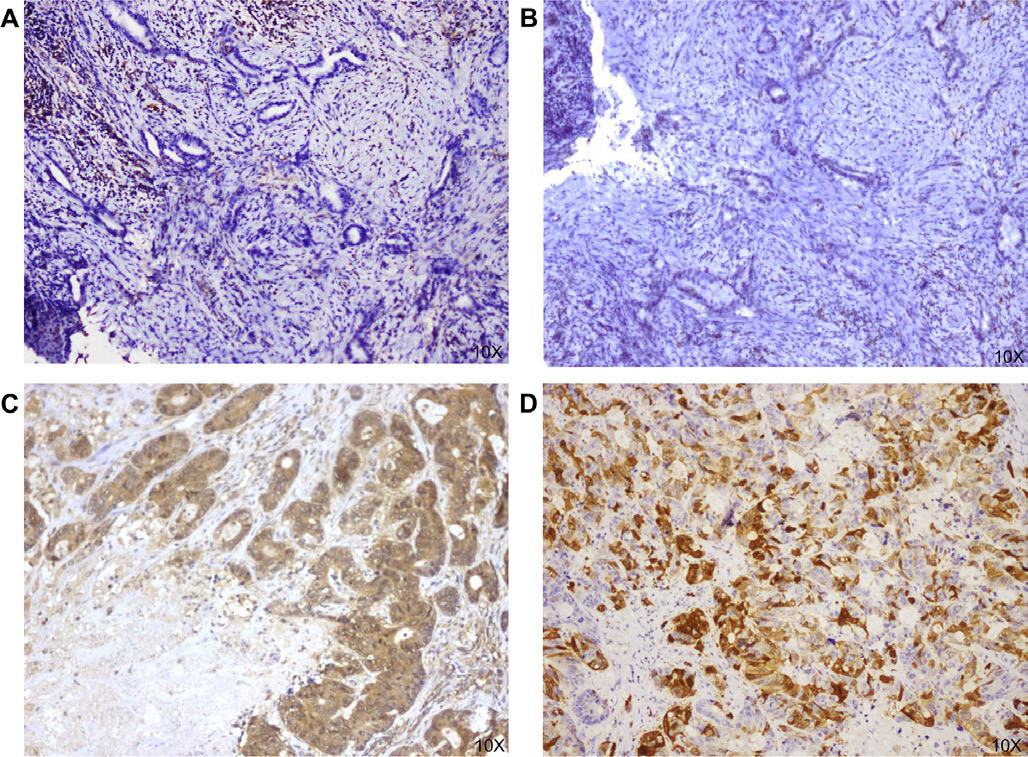
Immunohistochemistry (IHC) staining of two cases of metastatic colorectal cancer harbouring *MTAP* alterations other than gene loss. (A, B) Samples were collected from a patient in which an *MTAP-CDKN2B truncation* was identified by next-generation sequencing (NGS), in which both MTAP and p16 IHC revealed a complete lack of expression for both proteins, respectively. (C, D) Samples were collected from a patient in which *MTAP* M140V point mutation was identified by NGS, in which both MTAP and p16 protein expression was maintained, respectively. MTAP IHC staining was performed using a rabbit polyclonal anti-MTAP antibody (1:200 dilution, Pro-teinTech, Tucson, AZ). As a control, we also evaluated the expression of the protein p16 (the product of CDKN2A gene) using the p16, Clone:JC2, mouse monoclonal antibody (1:100 dilution, Gennova, Sevillia). Only samples with complete MTAP and/or p16 protein loss were considered MTAP deficient (IHC score 0).

**Table 2:** Clinical and molecular characteristics of GI malignancies exhibiting *MTAP* loss as compared with *MTAP* unaltered GI tumours from the TCGA PanCancer Atlas Studies

	*MTAP* loss	*MTAP* unaltered	P value
No of patients	128	1224	
Median age (IQR)	63 (56–72)	66 (57–74)	0.151
Sex (%)			0.039
Male	89 (69.5)	730 (59.6)	
Female	39 (30.5)	462 (40.2)	
NA	0 (0.0)	2 (0.2)	
Cancer type (%)			<0.001
Gastro-oesophageal	78 (60.9)	534 (43.6)	
Pancreatic	40 (31.3)	138 (11.3)	
Colorectal	6 (4.7)	520 (42.5)	
Biliary	4 (3.1)	32 (2.6)	
Tumour histology (%)			<0.001
Adenocarcinoma	95 (74.2)	1002 (81.9)	
Mucinous adenocarcinoma	2 (1.6)	75 (6.1)	
Signet ring carcinoma	4 (3.1)	80 (6.5)	
Squamous carcinoma	27 (21.1)	67 (5.5)	
Stage at diagnosis (%)			0.495
Non metastatic	82 (64.1)	931 (76.1)	
Metastatic	12 (9.4)	103 (8.4)	
NA	34 (36.2)	190 (15.5)	
Tumour mutational burden* (median (IQR))	2.5 (1.6–4.1)	3.3 (2.07–5.3)	<0.001
Microsatellite instability† (%)	1 (0.8)	164 (13.4)	<0.001
*CDKN2A/B* loss (%)	125 (97.7)	57 (8.1)	<0.001
*TP53* mutant (%)	84 (65.6)	703 (57.4)	0.044
*KRAS* mutant (%)	41 (32.0)	332 (27.1)	0.253
*BRAF* V600E mutant (%)	1 (0.8)	48 (3.9)	0.080
*APC* mutant (%)	6 (7.1)	435 (35.5)	<0.001
*ERBB2* amplification	21 (16.4)	92 (7.5)	0.001

* Nonsynonymous TMB.

† According to the MANTIS score with a threshold of 0.4.

GIgastrointestinalMANTISMicrosatellite Analysis for Normal-Tumor InStabilityNAnot availableTCGAThe Cancer Genome AtlasTMBTumour mutational burden

We performed survival analysis (online supplemental figure 4). Patients with *MTAP* loss showed unfavourable PFS (17.06 months, 95% CI 13.15 to 24.33 vs 42.31 months, 95% CI 36.03 to 63.42, p<0.001) and a trend towards worse OS (20.35 months, 95% CI 17.92 to NA vs 49.38 months, 95% CI 44.74 to 58.55, p=0.002). However, after performing a multivariate Cox proportional hazards model to investigate the impact of *MTAP* loss and different GI cancer types on patient prognosis, this result did not reach statistical significance (HR=0.79, p=0.097). Indeed, when we included the type of cancer in our model, we found significant differences in PFS between different cancer types, with significantly worse PFS for PC (HR=3.13, p<0.001), BTC (HR=3.11, p<0.001) and GEC (HR=1.89, p<0.001). Since we previously observed a different distribution of cancer types according to *MTAP* status with a higher frequency of more aggressive cancer types in the *MTAP* loss group, this uneven distribution may explain the observed difference in PFS between the two cohorts, rather than the *MTAP* classification itself. Indeed, there was no survival difference according to *MTAP* status when addressing different tumour types separately (figure 2A,B and online supplemental figure 5).

### Niguarda cohort analysis

We analysed NGS reports of 558 GI tumours. Sequencing failed for 50 samples, leaving an initial cohort of 508 GI cancer patients to be screened for *MTAP* alterations (329 CRC, 80 PC, 47 GEC, 36 BTC, 16 small bowel or other tumours).

*MTAP* alterations were found in 27 (5.3%) (online supplemental figure 1B). Characteristics of patients are reported in table 1. Overall, most patients (14/27, 51.8%) were metastatic at diagnosis. Adenocarcinoma was the most common histology (25/27, 92.6%). *MTAP* alterations were found in PC (12/80, 15.0%), GEC (4/47, 8.5%) and BTC (2/36, 5.5%), CRC (7/329, 2.1%), and others including small bowels (2/16, 12.5%) (online supplemental table 2). Homozygous *MTAP* loss was the main deleterious alteration retrieved (22/27, 81.5%), followed by mutations (5/27, 18.5%), although differences were noticed according to primary tumour histology.

All tumour types but CRC were characterised by gene loss as the only *MTAP* genetic alteration. In fact, all *MTAP* alterations other than gene loss were retrieved in CRC, that was indeed enriched with 5/7 alterations being mutations (three reported as pathogenic: splice site 34–1G>A, A191fs*6, MTAP-CDKN2B truncation; two as variants of unknown significance). One of these cases was hypermutated due to microsatellite instability. In all 5 *MTAP* mutant samples from CRC patients, we performed IHC analysis to investigate the genomic effect on protein expression. Only *MTAP-CDKN2B* truncation as assessed by NGS led to complete lack of MTAP expression (IHC 0), coupled with absence of p16 proteins expression (IHC 0) despite no CNA for *CDKN2A/B* was reported; this annotation suggests that both *MTAP* and *CDKN2B* were involved in a truncation event leading to absence of translation to the protein level, differently from other somatic variants with no or minor impact on the protein level. In all other cases, MTAP protein expression was retained (figure 3). We performed IHC in a case of CRC harbouring *MTAP* loss: although there was minimal heterogeneity in staining, 90%–98% of cancer cells did not express MTAP protein, as compared with intense and homogeneous protein expression in proficient cases.

Focusing on *MTAP* loss, all cases were microsatellite stable (100%) and *CDKN2A/B* deleted (100%). Given that PC was most represented in our cohort, we built a case–control study with a 1:2 ratio in PC patients (table 3). We found no significant difference in terms of clinical and molecular features apart from a higher prevalence of *CDKN2A/B* loss (100% vs 4.2%, p<0.001). Survival analysis did not show any difference for both PFS to first-line treatment and OS according to *MTAP* status (figure 2C,D).

**Table 3:** Clinical and molecular characteristics of *MTAP* loss versus *MTAP* unaltered pancreatic cancer patients from the Niguarda Cancer Center cohort

	MTAP loss cohort	MTAP intact cohort	P value
No of patients	12	24	
Median age at diagnosis (IQR)	63 (51–72)	65 (57–71)	0.750
Gender (%)			
Male	5 (41.7)	14 (58.3)	0.483
Female	7 (58.3)	10 (41.7)	
Stage at diagnosis (%)			0.725
Non metastatic	5 (41.7)	13 (54.2)	
Metastatic	7 (58.3)	11 (45.8)	
Tumour mutational burden (IQR)	1.26 (0.00–4.10)	2.52 (1.58–4.73)	0.210
Microsatellite instability (%)	0.0	0.0	NA
*CDKN2A/B* loss (%)	12 (100.0)	1 (4.2)	<0.001
*RAS* mutant (%)	23 (95.8)	12 (100.0)	1.000

MTAPmethylthioadenosine phosphorylaseNAnot available

## Discussion

In our study, we found that *MTAP* alteration prevalence in GI cancers ranges from 5% to 10% across various cancer types. We reported here that PC, GEC and BTC show the highest prevalence of *MTAP* loss among GI cancers (15%–22%, 9%–13%, 6%–11%, respectively). Conversely, CRC showed a lower incidence of *MTAP* alterations (2%), which were mostly mutations rather than gene loss. These results are in line with previous reports on the prevalence of *MTAP* alterations in GI cancers, confirming the enrichment of such molecular feature in upper-GI malignancies.^19^,^23^ In addition, our findings bring new knowledge regarding the very low prevalence of *MTAP* loss in lower-GI tumours (<1% in CRC).

Similar to other non-GI malignancies, *MTAP* and *CDKN2A/B* loss co-occurred in almost all cases. Moreover, in more than 50% of cases *MTAP* loss co-occurred with the loss of other genes located on chromosomal locus 9p21.3, thus leading to postulate that *MTAP* loss could be related to a large-scale chromosome deletion. We also investigated the potential clinical impact of *MTAP* mutations, that is, currently unknown. In our cohort, only one mutation out of five led to IHC protein loss, thereby limiting the potential use of targeted approaches in this setting.

Regarding prognosis, we found no association between *MTAP* loss and PFS and OS. Indeed, the higher prevalence of biliopancreatic cancers among *MTAP* loss cancers emerged as the actual driver of worse prognosis rather than the *MTAP* loss itself. Accordingly, in patients identified in the Niguarda series we did not observe any survival difference among *MTAP* loss and proficient cases. These data are in line with another recent study on BTC in which *MTAP* loss was not prognostic.^34^

Even if the overall results from both the cohorts are comparable, there are also some differences. First, the TCGA cohort is larger but less clinically detailed than our institutional cohort. Besides, in the TCGA nearly 75% of patients were affected by non-metastatic tumours, whereas all patients had metastatic cancers in our cohort. Finally, CRC was the most common histology in our cohort due to centre-specific enrichment, while GEC squamous tumours, in which *MTAP* loss plays an important role in carcinogenesis, accounted for only a minority of cases.^14^

Beyond *MTAP* loss, we found five out of seven *MTAP* altered CRC cases harbouring alterations other than gene loss. Given the availability of clinical trials targeting cancers with *MTAP* loss (online supplemental table 3), we performed IHC to test whether protein expression in these five cases was retained, but we found that only the *MTAP*-*CDKN2B* truncation led to protein loss, thus potentially having a clinical impact predisposing to sensitivity to MTAP targeted treatment strategies.

Our study has also limitations. First, no functional assessment of *MTAP* mutations has been performed. Second, the retrospective nature of the study and the limited sample size of some GI subset in the present cohort represent a further limitation. Third, the analysis was underpowered to distinguish small-effect size differences within individual tumour types, especially given the small numerosity of *MTAP* loss CRC and BTC. Finally, we acknowledge that all GEC in the Niguarda cohort were adenocarcinomas as a possible limitation, differently from the TCGA cohort. Indeed, since we retrieved molecular profiles data from GO40782/STARTRK-2 clinical trial screening (NCT02568267), according to physicians choice at our institution mostly adenocarcinoma patients rather than squamous cell cancer patients were screened for trial enrolment.

In conclusion, this is the first study investigating *MTAP* alterations prevalence in different GI cancers, also focusing on concomitant genetic alterations and addressing *MTAP* loss also at the transcriptomics and proteomics levels. We found that *MTAP* loss occurs almost exclusively in MSS tumours and it co-occurs with *CDK2NA*/*B* and/or other 9p21.3-located gene loss in almost all cases. *MTAP* loss was more common in upper-GI cancers (PC, BTC and GEC), compared with lower GI-cancers (ie, CRC). However, *MTAP* loss did not impact GI cancer prognosis. In CRC, we also identified *MTAP* alterations other than gene loss but, since protein loss was uncommon in these cases, it is unlikely that they may impact clinical outcomes and therapeutic targetability in GI cancers.

## supplementary material

10.1136/jcp-2023-209341 online supplemental file 1

## Data Availability

Data are available on reasonable request.
