# Supplementary material for: Clinicopathological characterisation of MTAP alterations in gastrointestinal cancers
Source: J Clin Pathol. 2024 Feb 13;78(3):e209341. doi: 10.1136/jcp-2023-209341 (PMC11874331; doi:10.1136/jcp-2023-209341)
Supplement: online supplemental file 1 [file jcp-78-3-s001.pdf]

Supplementary Figures

Supplementary Figure 1. Flow diagram of the study for the TCGA PanCancer Atlas cohorts (A) and the Niguarda Cancer Center cohorts (B).

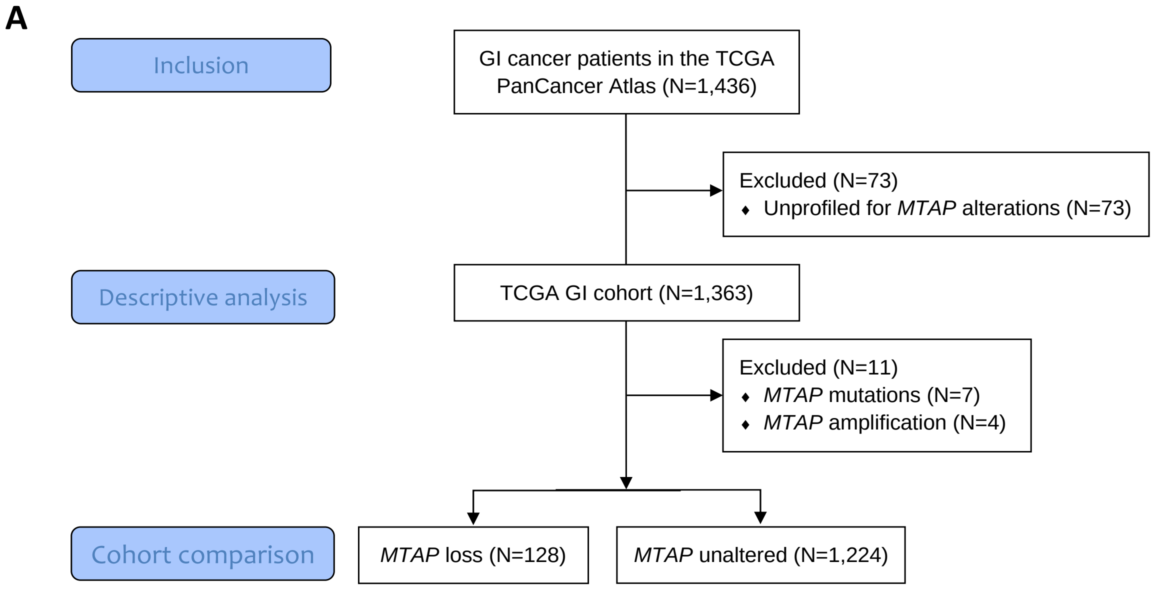

Keys. GI = gastrointestinal, N = numbers of patients.

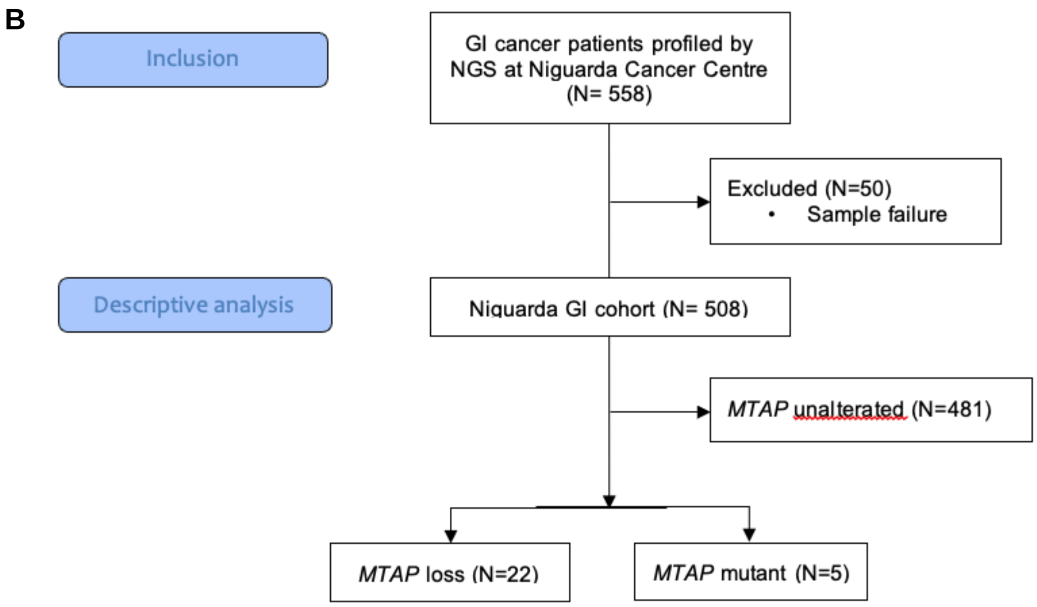

Keys. GI = gastrointestinal, NGS= Next generation sequencing, N = numbers of patients.

Supplementary Figure 2. Enrichment for gene deletions across chromosome 9p in the *MTAP* loss population from the TCGA PanCancer Atlas Studies.

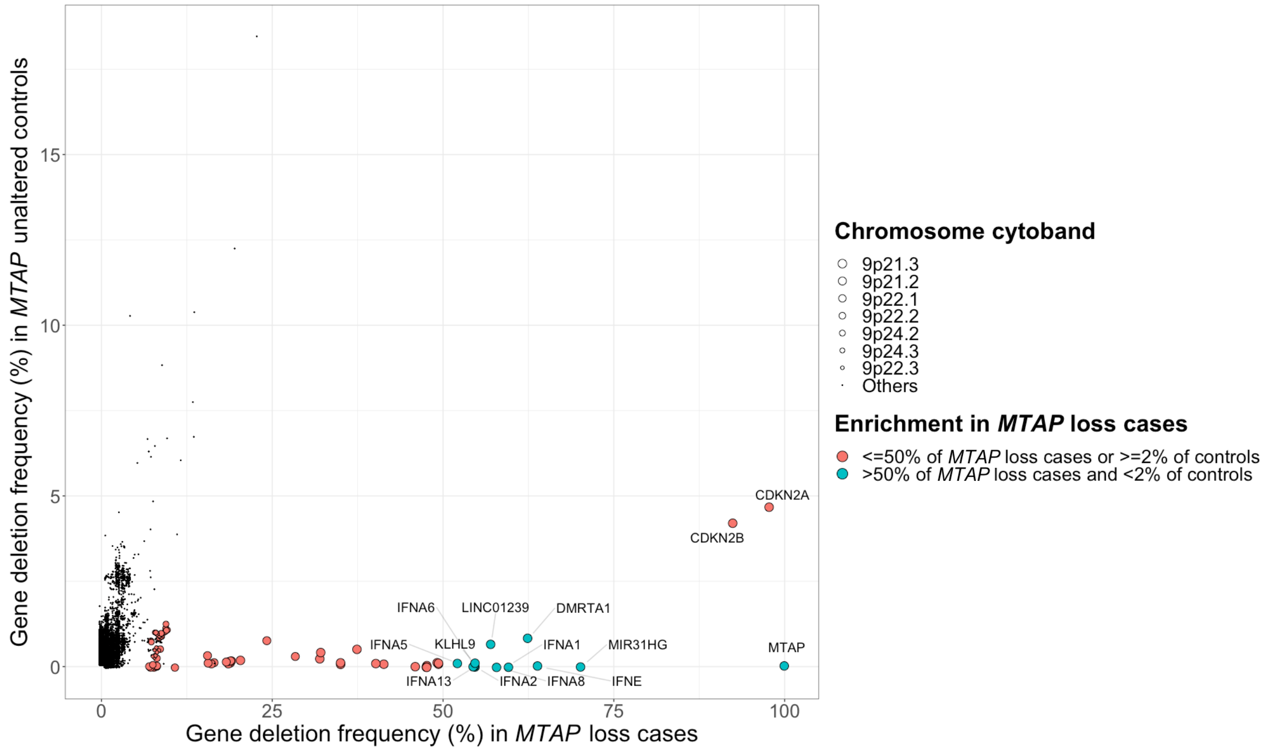

Supplementary Figure 3. Gene expression is significantly related to *MTAP* copy number in TCGA PanCancer Atlas Studies analysis.

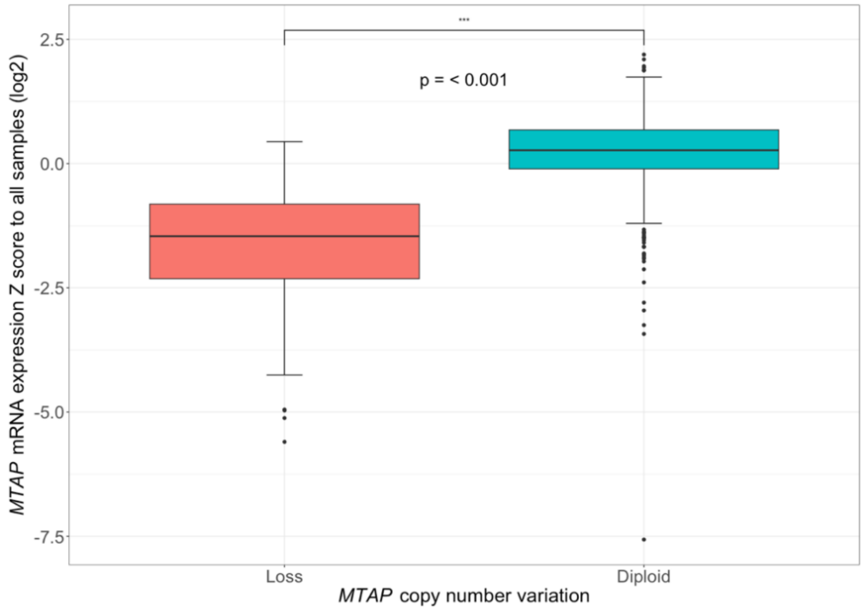

Box plot representing the mRNA expression levels of *MTAP* across different copy number variations. Expression values are first standardized by computing Z-scores, indicating how many standard deviations each data point is from the mean of the entire cohort. Then, data are transformed using a base-2 logarithm to represent fold changes to enhance visual representation.

Supplementary Figure 4. Survival analysis of *MTAP* loss *cases* vs *MTAP* unaltered controls from the TCGA PanCancer Atlas Studies.

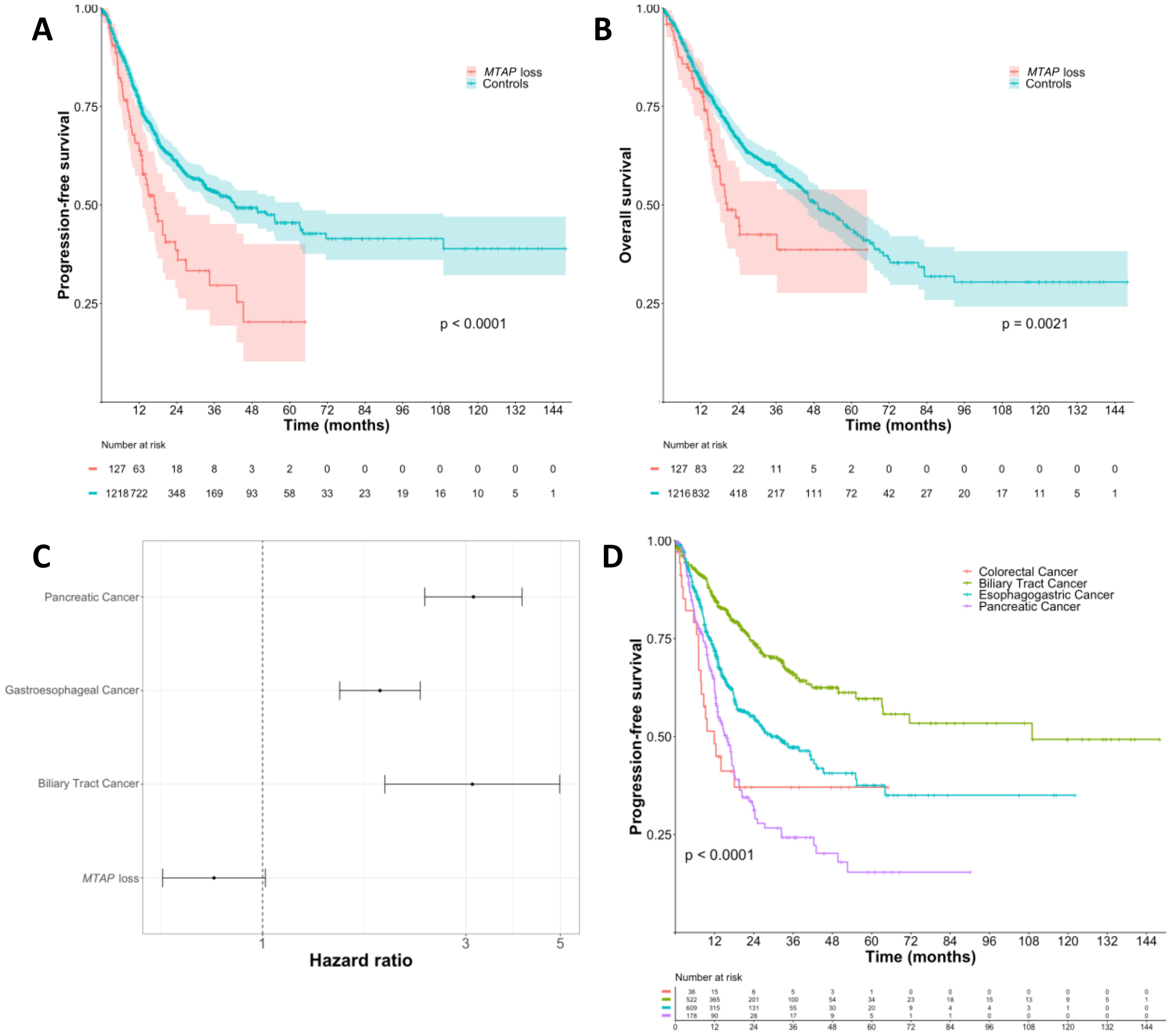

A-B. Progression-free survival analysis of *MTAP* loss vs *MTAP* unaltered cases. **C**. Cox proportional hazards regression models with multiple predictors (*MTAP* status and primary tumor site). **D**. Progression-free survival analysis according to primary tumor site.

Supplementary Figure 5. Survival analysis of *MTAP* loss *cases* vs *MTAP* unaltered controls for separate tumor types from the TCGA PanCancer Atlas Studies.

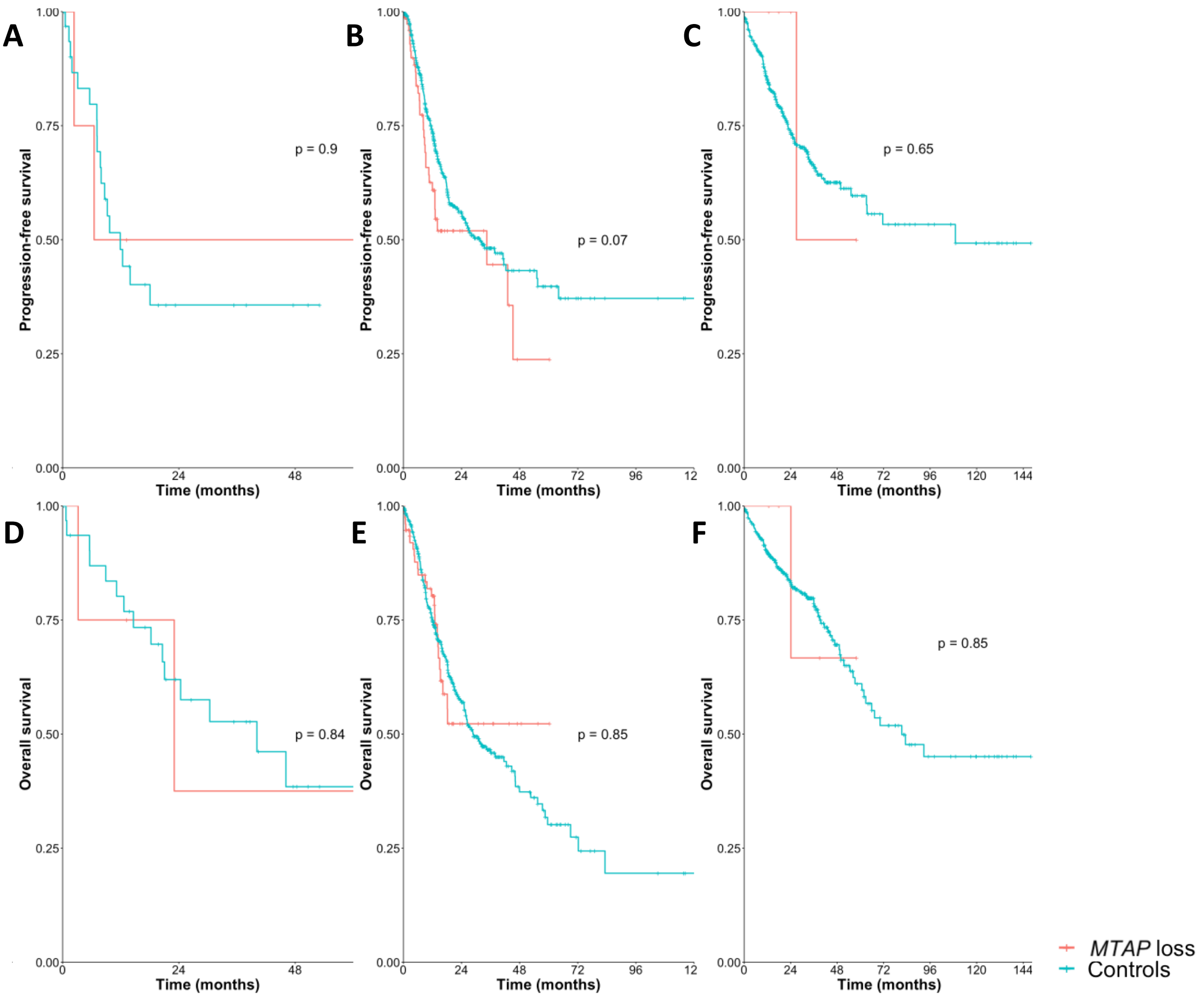

A-D. Progression-free survival analysis of *MTAP* loss vs *MTAP* unaltered cases, for pancreatic (A), biliary tract (B), gastroesophageal (C) and colorectal cancer (D). **E-H**. Overall survival analysis of *MTAP* loss vs *MTAP* unaltered cases, for pancreatic (A), biliary tract (B), gastroesophageal (C) and colorectal cancer (D).

Supplementary Tables

Supplementary Table 1. Patient characteristics and *MTAP* alteration prevalence in the overall cohort of GI cancer patients from the TCGA PanCancer Atlas Studies.

**Number of patients**	1363
**Age (median[IQR])**	66 [57-74]
**Sex (%)**	
Male	823 (60.4)
Female	538 (39.5)
NA	2 (0.1)
**Cancer type (%)**	
Gastroesophageal	616 (45.2)
Stomach	434 (70.5)^
Esophageal	182 (29.5) ^
Squamous carcinomas	95 (52.2) ^^
Adenocarcinomas	87 (47.8) ^^
Colorectal	532 (39.0)
Pancreatic	179 (13.1)
Biliary	36 (2.6)
**Tumor histology (%)**	
Adenocarcinoma	1106 (81.1)
Mucinous adenocarcinoma	77 (5.7)
Signet ring carcinoma	85 (6.2)
Squamous carcinoma	95 (7.0)
**Stage at diagnosis (%)**	
Non metastatic	1020 (74.8)
Metastatic	117 (8.6)
NA	226 (16.6)
**Tumor mutational burden* (median[IQR])**	3.2 [2-5.2]
**Microsatellite instability** (%)**	169 (12.4)
***MTAP* status (%)**	
Copy number loss	128 (9.4)
Copy number gain	4 (0.3)
Mutation	7 (0.6***)
Wild type	1224 (89.8)
***MTAP* loss prevalence by cancer type (%)**	
Gastroesophageal	78/616 (12.7)
Stomach	40/434 (9.2)^
Esophageal	38/182 (20.9)^^
Pancreatic	40/179 (22.3)
Colorectal	6/532 (1.1)
Biliary	4/36 (11.1)

*Nonsynonymous TMB

**According to the MANTIS score with a threshold of 0.4

***MTAP mutations were found in CRC (N=4) and GEC (N=3)

^ Percentages referred to the total of gastroesophageal carcinomas

^^ Percentages referred to the total of esophageal carcinomas

Supplementary Table 2. *MTAP* alteration prevalence by cancer type and subclassification of gastroesophageal in the Niguarda cohort.

**Number of patients**	508
***MTAP* alterations (%)**	27 (5.3)
***MTAP* alteration prevalence by cancer type* (%)**	
Gastroesophageal	4/47 (8.5)
Junctional	11 (23.4)^
Stomach	31 (66.0)^
NA	5 (10.6)^
Pancreatic	12/80 (15)
Colorectal	7/329 (2.1)
Biliary	2/36 (5.5)
Others	2/16 (12.5)
***MTAP* alteration type (%)**	
MTAP loss	22/27 (81.5)
MTAP mutation*	5/27 (18.5%)

*CRC was the only tumor type harbouring *MTAP* mutations, while all other alterations reported in the table were *MTAP* loss

^ Percentages referred to the total of gastroesophageal carcinomas

NA: not available data

Supplementary Table 3. Ongoing clinical trials targeting *MTAP* altered tumors.

Trial	Study type	Sponsor	Tumor site	Treatment	Mechanism of action	Status
NCT05975073	Phase 1/2	Amgen	Solid tumors	AMG 193 + IDE397	PRMT5 inhibitor + MAT2A inhibitor	Not yet recruiting
NCT05094336	Phase 1/2	Amgen	Solid tumors	AMG 193 +/- docetaxel	PRMT5 inhibitor	Recruiting
NCT04794699	Phase 1	Ideaya Biosciences	Solid tumors	IDE397 +/- CT	MAT2A inhibitor	Recruiting
NCT04089449	Phase 1	Prelude Therapeutics	Solid tumors<br>CNS lymphoma<br>High-grade gliomas	PRT811	PRMT5 inhibitor	Recruiting
NCT05275478	Phase 1	NEXT Oncology	Solid tumors	TNG908	PRMT5 inhibitor	Recruiting
NCT05732831	Phase 1/2	Tango Therapeutics	Solid tumors	TNG462	PRMT5 inhibitor	Recruiting
NCT05245500	Phase 1/2	Mirati Therapeutics Inc.	Solid tumors	MRTX1719	PRMT5-MTA inhibitor	Recruiting
NCT03435250	Phase 1	IRIS	Solid tumors<br>Lymphoma	AG-270 +/- taxane-based CT	MAT2A inhibitor	Terminated (Strategic reasons)
NCT00062283	Phase 2	NCI	Lung Cancer<br>Mesothelioma<br>Pancreatic Cancer<br>Sarcoma	L-alanosine	Purine Synthesis Inhibitor	Completed
NCT00075894	Phase 1/2	NCI	CNS tumors	L-alanosine	Purine Synthesis Inhibitor	Completed
NCT03666988	Phase 1	GlaxoSmithKline	Solid tumors<br>DLBCL	GSK3368715	PRMT inhibitor	Terminated (overall benefit-risk profile did not support continuation of the study)
NCT05312372	Phase 1/2	IRIS	Esophageal SCC	AG-270 + paclitaxel	MAT2A inhibitor	Withdrawn (Strategic reasons)
NCT00078468	Phase 2	Pfizer	CRC	AG-2037 (pelitrexol)	GARFT Inhibitor	Completed

Abbreviations: PRMT5: protein arginine methyltransferase 5; MAT2A: methionine adenosyltransferase-2a, MTA: Methylthioadenosine, CT: chemotherapy; DLBCL: diffuse large B-cell lymphoma; SCC: squamous cell carcinoma; CRC: colorectal cancer; GARFT: glycinamide ribonucleotide formyltransferase; IRIS: institut de recherches internationales servier, NCI: national cancer institute
